# Spatial analysis of elderly access to primary care services

**DOI:** 10.1186/1476-072X-5-19

**Published:** 2006-05-15

**Authors:** Lee R Mobley, Elisabeth Root, Luc Anselin, Nancy Lozano-Gracia, Julia Koschinsky

**Affiliations:** 1 RTI International, 275 Cox, 3040 Cornwallis Road, Research Triangle Park, NC 27709-2194, USA; 2 University of Illinois, Urbana-Champaign, 220 Davenport Hall, 607 South Mathews Avenue, Urbana, IL 61801-3671, USA

## Abstract

**Background:**

Admissions for Ambulatory Care Sensitive Conditions (ACSCs) are considered preventable admissions, because they are unlikely to occur when good preventive health care is received. Thus, high rates of admissions for ACSCs among the elderly (persons aged 65 or above who qualify for Medicare health insurance) are signals of poor preventive care utilization. The relevant geographic market to use in studying these admission rates is the primary care physician market. Our conceptual model assumes that local market conditions serving as interventions along the pathways to preventive care services utilization can impact ACSC admission rates.

**Results:**

We examine the relationships between market-level supply and demand factors on market-level rates of ACSC admissions among the elderly residing in the U.S. in the late 1990s. Using 6,475 natural markets in the mainland U.S. defined by The Health Resources and Services Administration's Primary Care Service Area Project, spatial regression is used to estimate the model, controlling for disease severity using detailed information from Medicare claims files. Our evidence suggests that elderly living in impoverished rural areas or in sprawling suburban places are about equally more likely to be admitted for ACSCs. Greater availability of physicians does not seem to matter, but greater prevalence of non-physician clinicians and international medical graduates, relative to U.S. medical graduates, does seem to reduce ACSC admissions, especially in poor rural areas.

**Conclusion:**

The relative importance of non-physician clinicians and international medical graduates in providing primary care to the elderly in geographic areas of greatest need can inform the ongoing debate regarding whether there is an impending shortage of physicians in the United States. These findings support other authors who claim that the existing supply of physicians is perhaps adequate, however the distribution of them across the landscape may not be optimal. The finding that elderly who reside in sprawling urban areas have access impediments about equal to residents of poor rural communities is new, and demonstrates the value of conceptualizing and modelling impedance based on place and local context.

## Background

### U.S. health insurance markets

This section is provided for readers with no background understanding of U.S. health insurance markets. The U.S. has many forms of private and public health insurance, with different levels of regulatory control and oversight. Persons over age 64 who have contributed to the Social Security (retirement income) System during their working years are entitled to Medicare health insurance; when they enroll they become Medicare beneficiaries. The majority of health insurance provided to people under age 65 is through their employers, and purchased from the private insurance industry. About 15 percent of the U.S. workforce does not have any form of health insurance, and they are called the uninsured. These are generally younger, lower wage workers in small companies, or marginal workers in companies that scale back employee benefits to save costs.

In an effort to modernize Medicare insurance, the Federal government has allowed private insurers who meet strict requirements to sell private insurance to the elderly, as a substitute for 'traditional' Medicare insurance. There are many forms of private insurance now being sold to the elderly, including some managed care plan types. Managed care plans restrict the choice of physicians and hospitals to include a set selected by the insurance plan, over whom the plan has more control in terms of utilization and expenditures. Managed care plans also provide preventive care and disease management services to their constituents, to keep them healthier and reduce their expenditures. Managed care plans are paid a set amount per person insured, per year, and are motivated to hold down costs so that, on average, they do not lose money. The alternative to any of these private plan options in 'traditional', or Fee-for-Service (FFS) Medicare. Traditionally, Medicare allowed physicians and hospitals to charge specific fees for specific services, so this type of insurance is known as Fee-for-Service (FFS) Medicare. Persons with FFS Medicare can use any doctor or hospital who agrees to accept the Medicare assigned fees for their services. There is no incentive in the FFS system to hold down costs, manage care, or provide preventive care or care management services to constituents.

There are two main types of managed care plans in the private market: Health Maintenance Organizations (HMOs), which require constituents to see particular doctors and use particular hospitals, or forfeit any coverage, and Preferred Provider Organizations (PPOs), which allow constituents to use outside physicians or hospitals at a cost, usually a small copayment. Managed care growth in the private sector has been effective in holding down growth in national health care costs. The Centers for Medicare and Medicaid Services, the federal government agency that oversees the Medicare program, has tried to interest seniors in voluntarily enrolling in Medicare managed care plans, to help contain the growth in Medicare expenditures. The growth of Medicare managed care plans (abbreviated MMC plans) has been variable over the past decade, and their penetration of the elderly insurance market has varied with enrollment and disenrollment behavior by the elderly. There has been no requirement that the elderly remain in managed care plans for any set length of time, and disenrollment occurs frequently, often to another managed care plan or back to FFS Medicare (where they become FFS beneficiaries). It is anticipated that, as the next generation of seniors ages into retirement, their greater familiarity with managed care through the workplace will make Medicare managed care more attractive to them than FFS Medicare.

In addition to the traditional FFS Medicare or Medicare Managed Care (MMC) insurance, many elderly buy supplemental insurance policies to cover prescription drugs or catastrophic expenses. These supplemental policies are known as MediGap plans, because they help fill gaps in the available health insurance coverage. Some Medicare beneficiaries are dual eligibles – covered by both Medicare (health insurance for the aged) and Medicaid (health insurance for the poor with chronic disabilities or end-stage renal disease). Dually eligible beneficiaries receive prescription drug coverage as part of their Medicaid insurance. During the period of this study (1998–2000) beneficiaries with FFS Medicare did not have any prescription drug coverage unless they had purchased supplemental insurance. About half of the Medicare managed care plans offered at least limited prescription drug coverage, but this study includes only those persons with FFS Medicare (we do not know whether they had supplemental MediGap or other drug coverage). Medicare managed care plan beneficiaries are excluded from the analysis because their plans are not required to submit their claims data to the Centers for Medicare and Medicaid, so there is no data source for use in the analysis. We include in the model Medicare managed care plan penetration and private insurance market competition variables because this competition can change the market climate, affecting and ways that medicine is practiced or the ways that people behave.

### ACSC literature

Access to care for the elderly continues to be a concern because the elderly may be more vulnerable to physical and financial constraints that would impede timely utilization of the healthcare services available to them. Impeded access can lead to under-utilization of primary care and preventive care services, which in turn may result in unnecessary hospitalizations, increased morbidity, and higher costs to the healthcare system than necessary.

The use of hospital admission rates for ambulatory care sensitive conditions (ACSCs) has become an established tool for analyzing access to care [1,2]. ACSCs are conditions for which good outpatient care can potentially prevent the need for hospitalization. High rates of hospital admissions for ACSCs may provide evidence of problems with patient access to primary healthcare, inadequate skills and resources, or a mismatch in services. Thus, ACSC hospitalization rates provide a practical way of evaluating primary care delivery and thereby identifying and targeting places where it may be possible to improve access and quality in the health care delivery system.

Studies have identified several factors that impact the rates of hospital admissions for ACSCs such as the aging of society, growth in out-of-pocket spending, an increasing level of frailty in the elderly, and enrollment in or disenrollment from managed care [3,4]. Having a regular source of care and continuity of care has been shown to significantly reduce the likelihood of hospitalizations and emergency room visits for ACSCs [5,6]. Limited access to care, such as living in an area with a shortage of health professionals or being uninsured, can also lead to higher ACSC admission rates [7].

Socioeconomic status, poverty, and race have been found to be correlated with ACSC rates [8-10]. Several studies have examined the associations between ACSCs and demographics using small areas of analysis (typically ZIP code) and have found that ACSCs are higher in low-income areas and areas with higher concentrations of racial and ethnic minorities [11,12]. The elderly population has not been studied much in this context, because they are thought to be relatively well-insured. However, Billings, Anderson, and Newman [11] found that socioeconomic class is important, even among the insured populations, concluding that barriers to accessing ambulatory care may extend beyond affordability to other factors, such as transportation or knowledge about how to engage the healthcare system. In this context, concern about increasing shortages of primary care physicians for Medicare beneficiaries, high turnover rates among the elderly in Medicare managed care (MMC) plans, lack of familiarity among the elderly with managed care practices, and rising rates of hospitalization for ACSCs have sharpened focus on the Medicare population [13,4,14]. The elderly may be especially vulnerable to impediments to travel and other factors characterizing the spatial interaction between people and their environments.

In this paper we carefully develop an access-to-care model that includes supply, demand, and ecological factors that serve to intervene along the pathways to healthcare utilization. We use data at a very low level of spatial aggregation – the Primary Care Service Area (PCSA) – which has not been used in previous ACSC research. We argue that the PCSA is the relevant market for examining ACSCs because these market boundaries are defined based on Medicare patient flows from home address to visit their primary care physicians. Meaningful associations between provider supply and outcomes should occur, and thus be examined, at this geographic scale. We obtained zip code level data for all Fee-For-Service (FFS) Medicare beneficiaries over a three-year period, 1998–2000. While this sample does not consider the entire Medicare population, the literature suggests that this FFS subgroup may be vulnerable because they lack any care coordination or management from their health *plan*. The FFS population is the subgroup with the greatest latitude in choosing providers, and is composed of members across the income spectrum. This subgroup of the Medicare population also spans the urban-rural continuum and provides insights that cannot be gleaned from studying the Medicare managed care population, who are urban-based.

### Conceptual model of access to preventive care services

Talen and Anselin [15] evaluate several different accessibility measures and state that the simplest 'container' approach (density of services per capita in a given area) can be misleading if the area is not well defined, i.e., there are significant flows of people from inside to outside or from outside the area to use services inside it. Another criticism is that it presumes that all people within the proscribed area are equally capable of accessing the services within it, which assumes away any spatial interaction that would either facilitate or impede access among specific population subgroups [16,17]. One way of addressing the problems inherent in the container approach is to develop market area 'containers' that represent, as accurately as possible, the actual geographic boundaries of the health care market. Health markets defined using patient flows are often better for analysis of access to care because they group small areas using variables that reflect utilization rather than imposing arbitrary spatial boundaries on the data.

The geographic markets we chose to use in this study, the Primary Care Service Areas (PCSAs), were developed using Medicare utilization data to represent geographic approximations of markets for primary care services received by the elderly [18]. We assume that these areas are the best approximation of the service areas in which the Medicare beneficiaries travel to receive ambulatory care, and are therefore the appropriate areal unit over which to construct aggregate rates for ACSC admissions.

**Figure 1:**
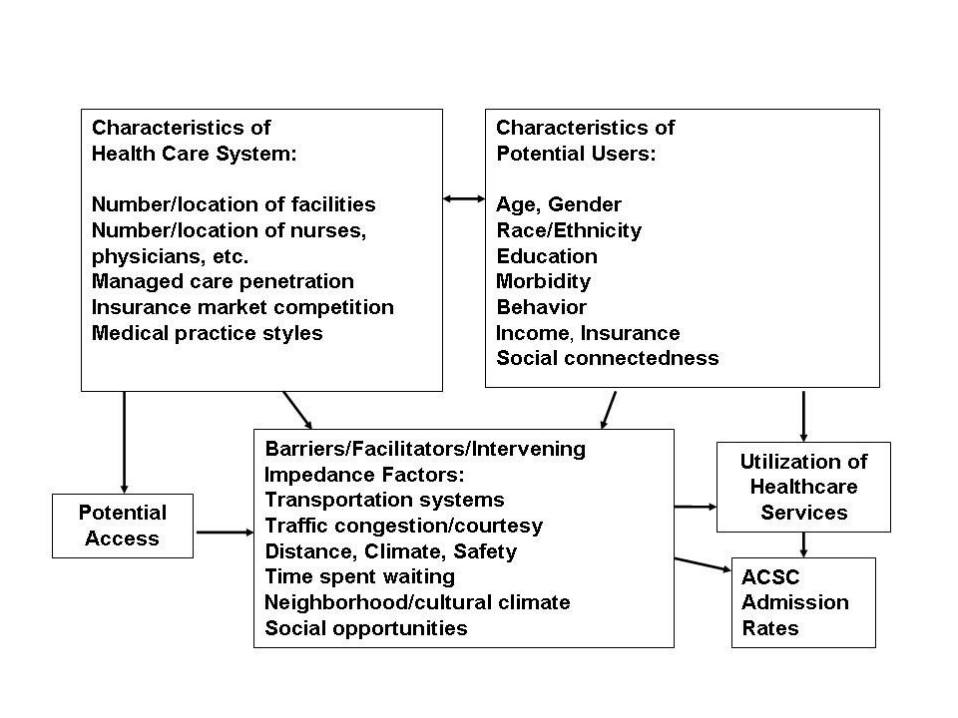
Spatial model of the utilization of healthcare services.

The theoretical framework we use in this paper combines traditional access to care and health service utilization models with a unique understanding of the spatial and geographic components of access and utilization. The Khan and Bhardwaj [19] model (Figure 1) employs a distinctly spatial view of human interaction with the environment and other structural and social aspects of the health care system. This "spatial interactions" approach considers how characteristics of the person (age, income, education, insurance), interact with characteristics of the health care system (location of providers, provider density, managed care penetration), and with intervening factors that can impact travel to or utilization of health facilities (transportation systems and traffic congestion, climate, safety, distance to facilities, time spent waiting for appointments and service, and neighborhood or cultural factors that may impact behavior and beliefs).

### Empirical model and expectations based on the literature

The dependent variable in this analysis is the rate of hospital admission for ACSCs by elderly with FFS Medicare insurance. The ACSC rate is a 3-year rate defined for each PCSA market, as follows. All hospital admissions for these ACS conditions during the interval 1998–2000, in each PCSA, were summed and then divided by the FFS Medicare beneficiary population in the PCSA in the middle year. The result was multiplied by 1,000 to produce a PCSA-level 3-year admission rate per thousand FFS beneficiaries. The conceptual model (Figure 1) contains several different groups of factors, and we include representatives of each category in our empirical model. Our expectations regarding how factors are associated with health outcomes (ACSC admission rates) are shaped by the literature, as follows.

### Demand factors

Socioeconomic status and race have been found to influence ACSC rates, as noted in the introduction. Local social and economic conditions may play a role in poverty dynamics. Poverty in a neighbourhood depends in part on fortunes of adjacent areas and who exactly is poor and where. We posit that elderly persons' poverty relative to poverty among the entire population may be important – i.e., elderly poor in a poor area are expected to have worse health access than elderly poor in an area where average income is higher. We construct a variable reflecting poverty among the elderly, and another reflecting the ratio of % elderly in poverty to % total population in poverty.

Poverty is higher in remote rural areas and in inner cities, but the rural elderly are much more likely to be poor than those living in urban areas. Thirteen percent of rural elders 60 years and older were poor in 2000, compared with nine percent of elders living in a metro area [20]. Thus we expect to find the most evidence of impeded access for the poor elderly who reside in rural areas. We interact the proportion of elderly in poverty with the proportion in rural areas to include in the model.

We also expect that elderly living among elderly in rural areas may have greater access impedance than elderly living among a population of mixed ages in rural areas. We construct a variable reflecting the relative isolation of the elderly by dividing the percent elderly in the area who are rural by the percent of the general population in the area who are rural. We expect that higher values of this ratio reflect greater isolation of elderly in rural areas, which is expected to impede utilization of healthcare and increase ACSC admission rates.

### Supply factors

A growing body of literature argues that the availability and mix of physician specialties in areas is important for health outcomes. Areas with fewer specialists but higher generalists per capita were found to have better health outcomes or quality of care [21,22]. Goodman [23] found that greater physician supply is associated with both higher area income and lower mortality rates, and argued that regional variations in health outcomes and physician supply will exist as long as there are differences across communities in economic status.

A long-standing tenet of state and federal physician workforce policy is that the provision of income supplements to physicians in rural areas will help attract physicians to these areas. Goodman [23] examined changes in physician settlement patterns over a 20 year period and found that there has been only a little change in the relative distribution of physicians across urban and rural areas. While the aggregate supply of physicians per capita grew 50 percent, most physicians located in urban areas where the supply per capita was already larger, and by 1999 there was still greater than 300 percent variation in physicians per capita across the 306 Hospital Referral Regions (HRRs) used for the study. HRRs are rather large geographic boundaries that reflect markets for referral-sensitive cardiovascular surgical procedures and neurosurgery. The HRR boundaries were derived based on flows from home address to where Medicare FFS patients were hospitalized. All eleven HRR regions with an undersupply of generalists in 1979 were lifted above this threshold by 1999. However, variation in need in smaller areas within HRRs, such as the Primary Care Service Areas (PCSAs), has been documented – which means that small local area shortages of physicians may still exist [18]. One study finds that policies aimed at increasing physician supply in rural areas have been successful [24]. Another finds that international medical graduates (IMGs) have disproportionately located in U.S. counties of greatest need, compared to U.S. medical graduates [25].

Other literature examines the importance of non-physician clinicians in health care [26,27]. States with the highest ratios of non-physician clinicians (nurse practitioners, physician assistants, and advanced practice nurses) to physicians were also the most rural. All things considered, the very recent findings from the 2000–2001 Community Tracking Survey, that rural America's healthcare access and quality is now as good or better than urban areas, is not too surprising [28]. However, this study was nationally representative, not focused on access to care by the elderly *per se*.

### Intervening factors

The Reschovsky and Staiti study [28] interviewed both patients and physicians, and provides considerable insight regarding differences in physical accessibility across the urban-rural continuum. The nationally representative survey was fielded in urban, suburban, and remote rural regions. Persons in remote rural regions had significantly longer travel times to see physicians and specialists than persons in metropolitan areas (2 minutes longer to see a physician and 34 minutes longer to see a specialist). However, persons in isolated rural areas were significantly less likely to say they couldn't get an appointment soon enough, and only persons in adjacent (suburban) metropolitan areas complained more about transportation *problems*.

We include in our model a variable reflecting the percent of the workforce who travel more than 60 minutes to work as an intervening variable reflecting commuter traffic and travel impedance for the elderly. This variable reflects urban sprawl, because residents of the sprawling suburbs are the most likely to have long daily commutes to and from work, clogging the local roadways. We expect that the elderly living in regions with greater numbers of long commuters will have more difficulty driving the roads.

Managed care prevalence in the market can also impact the climate in which the elderly seek care. The availability of managed care plans for the elderly could improve elderly access to and utilization of preventive care services, if the Medicare managed care plans fulfill their promise – more specifically, the management and coordination of care. A growing body of literature has found that Medicare beneficiaries in HMOs receive more preventive services and have better outcomes than their FFS counterparts. Rizzo [29] found that Medicare beneficiaries enrolled in HMOs received significantly and substantially higher preventive care services than beneficiaries in traditional FFS.

Other research has found that managed care may improve access for the poor and traditionally underserved [30]. In the context of the elderly population, because Medicare managed care has only penetrated urban areas, we expect that the poor elderly in urban areas will have managed care advantages not available to their poor rural counterparts.

If managed care does improve access to care for the elderly, then the elderly not enrolled in managed care – such as the FFS population we examine here – may be especially vulnerable to physician shortages. The wealthier elderly in FFS Medicare often hold supplemental coverages, perhaps enhancing their access to primary care physicians and other health services such as prescription drug coverage [31]. The elderly in FFS Medicare who don't hold supplemental insurance coverage are expected to be more vulnerable to physician shortages and impeded access to care.

We include in our model variables reflecting current Medicare HMO, and current private sector HMO and PPO penetration. We also include changes in these over recent time which reflect competitive conditions in managed care markets. Other competitive factors such as insurance industry concentration or prevalence of employer-sponsored retirement plans can also impact the climate in which the elderly seek care. We include state-level variables reflecting the private insurance market's concentration, the prevalence of employer-sponsored retirement insurance, and the average price of a standard MediGap plan in the area.

### Expectations

Many of the studies noted above regarding the relationships between physician supply, income, health services, and outcomes were not able to control well for disease severity. In our study we have a direct measure of disease risk for each beneficiary (aggregated across beneficiaries to the PCSA level) and the proportion in an area that are in the upper quintile of the risk distribution, as well as other clinical information such as whether diabetic or has end-stage renal disease, and age. Using the PCSA level of spatial aggregation as the primary unit of analysis, we are able to test several hypotheses regarding associations between the multiple factors in the spatial access model and health outcomes. Holding person-specific factors constant, we hypothesize that:

1. Availability of more physicians per capita is expected to be negatively correlated with ACSC admission rates.

2. Places with greater numbers of elderly visits (per capita) to doctors and health clinics are expected to have lower ACSC rates.

3. Poverty among the elderly is expected to be positively associated with ACSC admission rates, but more so in remote rural regions.

4. Greater managed care penetration is expected to be associated with lower rates of ACSC admissions.

5. Availability of supplemental coverage (in addition to or instead of Medicare) in an area is expected to be negatively associated with ACSC admission rates.

6. Urban sprawl as measured by long commutes for the local workforce is expected to be positively associated with ACSC admission rates.

## Results

### Empirical findings

Variable descriptions are presented in Tables 1 and 2, and construction of variables is described in the Methods section, below. Spatial regression methods and the rationale for using the spatial spillovers model are presented in the Methods section, below, with a discussion of what spatial spillovers are and why they might manifest themselves and cause problems in regression. Regression results are presented in Table 4, where both heteroskedasticity-consistent OLS and spatial lag regression models are presented. Table 3 presents sample statistics, including the mean, median, standard deviation, minimum, and maximum for each variable. Variable descriptions (Tables 1 and 2) reveal that there are many different units of measurement in the analysis – rates per thousand, proportions, percents, dollars, ratios, or visits per person.

**Table 1:** Description of Population and Demographic Variables

**Variable**	**Description**	**Source and primary level**
**Medicare FS Beneficiary Data**		
**ACSCRATE**	Count of admissions for any of 11 ACSCs, per 1,000 Medicare FFS beneficiaries, in the ZIP code of residence	CMS FFS MEDPAR claims, 1998–2000, ZIP code of residence
**XMEN**	Proportion of FFS beneficiaries in the ZIP code of residence that are male	"
**XDUAL**	Proportion of FFS beneficiaries in the ZIP code of residence that are dually eligible for Medicare and Medicaid	"
**XBLACK**	Proportion of FFS beneficiaries in the ZIP code of residence that are black	"
**XOTHER**	Proportion of FFS beneficiaries in the ZIP code of residence that are other races than white or black	"
**XDIED**	Proportion of FFS beneficiaries in the ZIP code of residence that died	"
**XOLDER**	Proportion of FFS beneficiaries in the ZIP code of residence that are over 80	"
**RISK**	Median PIP_DCG risk score for FFS beneficiaries in the ZIP code of residence	"
**HIQUINT**	Proportion of FFS beneficiaries in the ZIP code of residence that are above the median in PIP_DCG risk score	"
**XDIAB**	Proportion of FFS beneficiaries in the ZIP code of residence that are diabetic	"
**Demographic Census data**		
**XELDERPOV**	Proportion of elderly in the census tract with 1999 income below the poverty level	US Census, census tract
**POVRATIO**	Ratio of proportion elderly in poverty to proportion general population in poverty	"
**XTRURELD**	Proportion elderly in the county who reside in rural census tracts	"
**RURATIO**	Ratio of proportion elderly in rural census tracts to the proportion of total population in rural census tracts	"
**XLIVALONE**	Proportion of elderly who live alone	"
**XLCOMUTE**	Proportion of the workforce that commute longer than 60 minutes to work, each way	"
**XPOORNE**	Proportion of the elderly population who speak little or no English	"
**PDENSITY**	Population per square mile	"

**Table 2:** Description of Other Variables Used in the Analysis

**Variable**	**Description**	**Source and level**
**Facilities and Utilization Data**		
**BEDREHAB**	Number of beds in a PPS exempt rehabilitation unit of a hospital	CMS Provider of Service (POS), ZIP code
**VISITS**	Medicare Part B and outpatient primary care visits or ambulatory care visits, per Medicare Part B and outpatient beneficiary resident in the PCSA, plus number of primary care visits to rural health clinics or federally qualified health clinics per Medicare outpatient beneficiary resident in the PCSA	CMS CECS DENOM & Part B & Outpatient, PCSA
**Practitioner Data**		
**TOTDOCS**	Count of clinically active specialists and primary care physicians per 1,000 population	AMA/AOA Masterfiles, PCSA
**ALT_DOC**	Ratio of the count of nonphysician clinicians to physicians, by state, 1995	Cooper et al, 1998b; state
**IMG_RATIO**	Ratio of the count of international medical graduate physicians to clinically active specialists and primary care physicians	AMA/AOA Masterfiles, PCSA
**Market Conditions Data**		
**MCPENE00**	MMC PENETRATION of Medicare beneficiaries in 2000	CMS Geographic Service Area File, county
**CINCREASE**	Binary indicator of an increase in competition among the MMC plans available, between 1998–2000, from inverse Herfindahl index	CMS Geographic Service Area File, county
**XHMO00**	Penetration of state population by commercial HMOs, 2000	InterStudy, state
**XHMODIF**	Change in penetration of state population by commercial HMOs, 1994 – 2000	InterStudy, state
**XPPO00**	Penetration of state population by commercial PPOs, 2000	InterStudy, state
**XPPODIF**	Change in penetration of state population by commercial PPOs, 1994 – 2000	InterStudy, state
**SHRLARG3(%)**	Percent market share of the largest three commercial group market insurers in 1997–2001	Academy for Health Services Research and Health Policy, state
**ECOV97_9(%)**	Percent of elderly who have employer-sponsored health insurance	American Association of Retired Persons (AARP), state
**PRICE00A($)**	Annual premium for AARP's MediGap Plan A, 2000	RTI analysis of AARP MediGap premiums, state

**Table 3:** Sample Statistics

	Mean	Median	Standard Deviation	Minimum	Maximum
ACSCRATE	99.55	94.19	35.22	0.00	468.29
XMEN	0.41	0.41	0.03	0.29	0.68
XDUAL	0.14	0.11	0.10	0.00	0.76
XBLACK	0.06	0.01	0.12	0.00	0.99
XOTHER	0.03	0.01	0.07	0.00	0.94
XDIED	0.06	0.06	0.01	0.00	0.13
XOLDER	0.29	0.29	0.05	0.00	0.59
RISK	0.82	0.81	0.08	0.54	1.72
HIQUINT	0.37	0.36	0.06	0.05	0.73
XDIAB	0.15	0.14	0.06	0.00	0.85
XELDERPOV	0.11	0.09	0.06	0.00	0.57
POVRATIO	0.90	0.84	0.36	0.00	4.13
XTRURELD	0.70	0.82	0.33	0.00	1.15
RURATIO	2.23	1.58	3.25	0.73	66.00
XTRURELD* XELDERPOV	0.08	0.07	0.07	0.00	0.46
XLIVALONE	0.28	0.29	0.04	0.08	0.58
XLCOMUTE	0.08	0.07	0.05	0.00	0.41
XPOORNE	0.02	0.00	0.06	0.00	0.84
PDENSITY	915.45	62.18	4526.54	0.32	101144.30
BEDREHAB	3.96	0.00	14.09	0.00	213.00
VISITS	9.83	9.45	2.23	0.00	25.23
TOTDOCS	0.608	0.549	0.454	0.00	6.93
ALT_DOC	0.12	0.12	0.05	0.04	0.40
IMG_RATIO	0.45	0.27	0.95	0.00	38.34
IMG_RATIO* XTRURELD* XELDERPOV	0.03	0.01	0.13	0.00	4.98
MCPENE00	0.09	0.01	0.13	0.00	0.55
CINCREASE	0.16	0.00	0.35	0.00	1.00
XHMO00	0.24	0.22	0.13	0.01	0.54
XHMODIF	0.54	0.51	0.46	-1.07	2.00
XPPO00	0.20	0.18	0.08	0.03	0.47
XPPODIF	0.04	0.05	0.12	-0.32	0.40
SHRLARG3	53.15	53.00	14.99	23.00	92.00
PRICE00A	837.26	816.24	97.96	665.76	1168.73
ECOV97_9	32.27	32.00	6.80	19.10	52.80

**Table 4:** Regression Results from Three Models, n = 6455 PCSA-level observations

	**OLS Model^1^**		**Spatial Lag Model^2^**		**IV Spatial Lag Model^3^**	
**Variable**	**Coeff**	**St. Error**	**Coeff**	**St. Error**	**Coeff**	**St. Error**

**XMEN**	-198.82*	15.636	-119.50*	12.728	-142.79*	14.530
**XDUAL**	-359.43*	18.393	-293.70*	12.131	-284.88*	15.777
**XBLACK**	-33.69*	4.601	-22.69*	3.283	-21.65*	3.948
**XOTHER**	**-40.55**	17.595	**-11.49**	7.397	-61.30*	12.695
**XDIED**	672.79*	55.498	636.97*	38.625	562.14*	47.204
**XOLDER**	-648.11*	23.509	-482.83*	16.024	-476.86*	21.749
**RISK**	**12.83**	5.529	**0.99**	5.458	**5.34**	4.709
**HIQUINT**	845.10*	31.274	678.99*	20.579	661.21*	27.717
**XDIAB**	50.47*	9.773	43.41*	4.857	46.42*	8.126
**(1) XELDERPOV**	**24.45**	18.154	**21.58**	14.155	**21.25**	15.224
**POVRATIO**	-2.36*	0.910	**-0.64**	0.834	**-1.11**	0.780
**(2) XTRURELD**	-4.89*	1.972	**-1.13**	1.870	**-0.99**	1.716
**RURATIO**	**0.09**	0.076	**0.14**	0.087	**0.04**	0.068
(1)*(2)	84.67*	18.235	**33.17**	14.061	43.29*	15.591
**XLIVALONE**	**-3.24**	10.477	**-11.31**	8.078	**4.39**	8.970
**XLCOMUTE**	72.25*	6.889	43.14*	5.772	56.98*	6.039
**XPOORNE**	-90.97*	19.722	-76.49*	9.753	**-29.80**	14.875
**PDENSITY**	0.00*	0.000	0.00*	7.557	0.00*	0.000
**BEDREHAB**	-0.08*	0.016	-0.08*	0.020	-0.08*	0.014
**VISITS**	**-0.50**	0.212	-0.54*	0.128	-0.63*	0.174
**TOTDOCS**	**1.79**	0.819	**0.49**	0.626	**0.92**	0.725
**ALT_DOC**	-87.09*	8.399	-31.05*	7.246	-38.23*	7.780
**(3) IMG_RATIO**	4.42*	0.993	3.68*	0.577	4.73*	0.841
(1)*(2)*(3)	-23.64*	7.593	-19.33*	4.273	-22.40*	6.270
**MCPENE00**	**-5.98**	3.289	-10.28*	3.174	-7.52*	2.909
**CINCREASE**	**-0.62**	0.786	**-1.22**	0.826	**-1.09**	0.690
**XHMO00**	-13.62*	3.147	**-3.04**	2.864	**-4.91**	2.748
**XHMODIF**	-2.36*	0.842	-2.45*	0.666	-2.97*	0.717
**XPPO00**	-38.77*	5.049	-22.09*	4.714	-23.20*	4.405
**XPPODIF**	20.50*	3.505	14.83*	3.115	11.51*	2.998
**SHRLARG3**	-0.07*	0.021	**-0.02**	0.020	-0.06*	0.018
**PRICE00A**	0.02*	0.004	0.01*	0.003	0.01*	0.004
**ECOV97_9**	-0.32*	0.052	**-0.12**	0.047	-0.19*	0.048
**W_ACSC**			0.42*	0.012	0.33*	0.021
**N**	**6,475**		**6,475**		**6,475**	
**GOF measure^4^**	**0.7743882**		**0.774075**		**0.775249**	
**Log Likelihood**			**-28748.9**			

^1^Model estimated using SYSTAT with heteroskedasticity-corrected standard errors. ^2^Model estimated using GeoDa. ^3^Model estimated using PYTHON programming in R, with heteroskedasticity-corrected standard errors. ^4 ^To make this comparable across models, we report the correlation between observed ACSC rates and predicted values from each model. For the lag or IV model, predictions properly account for endogeneity of the lag term or for the degrees of freedom lost in instrumentation. *These coefficients are statistically significant at the 0.01 level.

To make the interpretation of results simpler and more comparable across variables, we present the discussion of coefficient effects in terms of standard deviation changes in their variables. A standard deviation change is a meaningful amount, as the area under a variable's distribution between the mean and 1 standard deviation above the mean is about 25 percent of the probability. A single unit change is often not meaningful (i.e., a 1 percent or one dollar or one additional doctor per capita) and rather than use an arbitrary amount of change that varied across variables, we use a consistent amount of increase – 1 standard deviation's worth in the variable's distribution – which is comparable across variables. In discussion of the results, the word 'significant' denotes statistical significance, which may occur even when impacts are so small as to have little practical importance.

Because the distributions of the dependent variable (and model errors) are quite skewed and there are many observations, we estimated an instrumental variables (IV) variant of the spatial lag model, in addition to the usual Maximum Likelihood Estimator (the MLE is more powerful when the assumption of normality is true) [32]. The MLE model estimates the spatial lag term as an endogenous variable within a simultaneous equations system. The IV model uses two stage least squares with spatially lagged right-hand side variables as instruments for the (endogenous) spatial lag term, with the White correction to standard errors for robustness against heteroskedasticity. We present all three models for comparative purposes, to demonstrate the robustness of the findings. The three models agree on the algebraic sign (positive or negative) of all statistically significant coefficient estimates (those with p value ≤ 0.01). The estimated coefficient of the spatial lag term (ρ, see equation 1) is significant in both of the spatial models, and reflects the extent of spatial spillovers across neighboring PCSAs due to common medical practice styles, resource constraints, or health behaviours.

The presence of a significant spatial lag parameter means that the parameters for all explanatory variables in the OLS model are overstated estimates of their *marginal *impacts, due to spatial multiplier bias. The OLS parameters reflect the compounded effect of the covariate (inclusive of spillovers), rather than the marginal effect (net of spillovers)[33]. An interpretation of the spatial lag parameter is that some of the impact of a particular covariate on ACSC admission rates is attributed to practice style or behavioral spillovers among residents and physicians in neighboring PCSAs. The magnitude of this spillover is directly proportional to the spatial lag parameter estimate. A significant lag parameter suggests that there is a regional pattern to behavior that is larger than the individual PCSA. With a lag parameter estimate of 0.33, every 1 standard deviation change in a covariate derives about half its impact from these spillovers or commonalities in behaviors (the spatial multiplier is 1/(1-ρ)). Failure to account for the redundancy or commonality in behaviors through muting these indirect effects leads to inflation of about 50 percent in the estimated *marginal *impact of the covariate on ACSC admission rates. If the compound effect is of interest, rather than the marginal one, this can be derived by multiplying the spatial lag model parameters by 1/(1-ρ), which is a multiplier of about 1.50. The OLS estimates are close to this magnitude of effect.

We focus the rest of the discussion on the spatial lag model estimated using instrumental variables. The person-specific factors all have quite significant associations with the outcomes. A one standard deviation (0.10, or 10 percent, see Table 3) increase in the proportion who are dually eligible (XDUAL) is associated with about 29 fewer ACSC admissions per thousand FFS beneficiaries (0.10 *-284.88 = 29). A one-standard-deviation (ten percent) increase represents about a 71 percent increase from the mean of 14 percent (0.71*0.14 = 0.10). This finding is interesting because it suggests that, holding disease severity constant, the supplemental coverage provided by Medicaid greatly enhances preventive care services utilization. This is not surprising, because low-income seniors with Medicaid have prescription drug coverage, which requires physician or clinic visits to obtain prescriptions. As noted below, higher outpatient visit rates to physicians or clinics are associated with lower ACSC rates.

Places with one standard deviation (0.05 or five percent, see Table 3) higher proportions of older elderly (XOLDER) have about 24 fewer admissions per thousand persons (0.05*-476.86 = -24). Holding disease characteristics of areas constant statistically, places with higher concentrations of octogenarians are apparently filled with healthier survivors, such as found in some preferred retirement enclaves. Using the query feature in our GIS, we located on a map PCSAs where more than 40 percent of the elderly were over age 80. There were 67 such PCSAs, and 70 percent of them had below-average ACSC rates. Ten were in Florida and of these, 7 had below-average ACSC rates. The two places with over 50 percent of elderly over age 80 were in Florida with lower-than-average ACSC rates.

A one standard deviation increase in the proportion who are in the highest quintile of the severity risk score distribution (HIQUINT) is associated with about 40 more ACSC admissions per thousand FFS beneficiaries. These numbers are large, about the same magnitude as a one standard deviation change in the ACSC rates themselves. Thus it is important to hold constant statistically these person-specific factors so that ACSC admissions attributable to residual variation can be explained by other factors.

The next block of variables is the demographic conditions in the PCSA of beneficiary address. Beneficiaries living in PCSAs where proportionately more elderly live in poverty (XELDERPOV) are not significantly more likely to be admitted for an ACSC, and when the elderly poverty rate is higher than that of the general population (POVRATIO) no significant association is found. Similarly, places with greater proportions of elderly in rural census tracts (XTRURELD) do not have significantly higher ACSC rates, even when the rural population is dominated by elderly (RURATIO). However, places with higher proportions of rural elderly who are also impoverished (the interaction variable XTRURELD*XELDERPOV) do have significantly higher ACSC rates, as expected. Because of concerns about potential multicollinearity, we checked the correlation between these two variables and found it to be lower than one might expect; 0.275. This is not large enough to cause multicollinearity problems. However, if the interaction is omitted from the model, XELDERPOV picks up its effect and the coefficient estimate almost quadruples. We conclude that it is not poverty per se, but rural poverty that seriously impacts ACSC rates. Unlike the poor elderly in urban areas, these rural residents do not enjoy the beneficial spillovers from managed care practices. A one standard deviation increase in the proportion of rural elderly who are impoverished increases ACSC admissions by 3 admissions per 1,000 FFS beneficiaries in the area.

Sprawling places where more of the working population commutes longer than 60 minutes each way to work have higher ACSC rates, reflecting transportation impedance for the elderly. About 3 additional admissions per 1,000 FFS beneficiaries in the area can be attributed to a one standard deviation increase in XLCOMUTE. Thus suburban sprawl is about equivalent to living in rural poverty in terms of the magnitude of association with ACSC admissions. This finding contributes to a growing literature on sprawl and adverse health outcomes [34-37].

The next block of variables represents facility availability and utilization. Rehabilitation beds are important for post-acute care among the elderly, and these beds have been subject to curtailed reimbursements following the Balanced Budget Act of 1997 [38]. Good post-acute care can contribute to better health and the functional ability to maintain one's health through activities of daily living (such as visits to providers). We find that rehabilitation bed availability is associated with significantly lower ACSC rates. Next, regarding utilization of healthcare visits to clinics and providers – higher outpatient visit rates to physicians or clinics is associated with modest but significantly lower ACSC rates (about 1.4 fewer ACSC admits per thousand for areas with a one standard deviation higher visit rate).

We found from preliminary regressions, where we used disaggregated physicians and visits (into different types for use) as independent variables, that coefficient estimates were unstable. This resulted because the physician groups and visit types were very highly correlated with one another. Aggregating specialists and generalists into a single physician variable (TOTDOCS) and four different visit types into a single visits variable (VISITS) solved the multicollinearity problem (their simple Pearson correlation is: -0.13). Physician availability (TOTDOCS) has no statistically significant association, which is not what we expected to find. However, areas with a higher proportion of non-physician clinicians to physicians (ALT_DOC) have significantly lower ACSC admission rates. A one standard deviation increase in the ratio is associated with about 2 fewer ACSC admissions per thousand FFS beneficiaries in the area. By linear extrapolation, two standard deviations is associated with about 4 fewer admissions. There are nine states with ratios higher than 2 standard deviations from the mean, and all are quite rural. Thus alternative providers seem to be filling a needed role providing primary care services in remote rural areas. These non-physician clinicians include nurse practitioners, physician assistants, and advanced practice nurses whose ability to provide autonomous patient care varies widely across geography [26,27].

The availability of more international medical graduates per U.S. trained physicians (IMG_RATIO) is also associated with significantly better outcomes, but only in the rural areas where the elderly are poor. The independent effect of IMG_RATIO is about 4.5 *more *admissions per one standard deviation increase in the variable, across all types of areas. But the estimated coefficient on the interaction (IMG_RATIO*XTRURELD*XELDERPOV) suggests that in poor, rural places a standard deviation increase in the IMG ratio is associated with about 3 *fewer *admissions per thousand (0.13*-22.4 = -3). Using a multivariate query in our GIS, we located 17 PCSAs where the values for XELDERPOV, XTRURELD, and IMG_RATIO were 1 standard deviation or more above their means (these places were across the southeastern US: Maryland, West Virginia, Kentucky, South Carolina, Missouri, Alabama, Arkansas, and Texas. So there are not very many rural places with impoverished elderly and where the ratio of IMGs to U.S. trained physicians is high, but where they do exist, there is a beneficial effect on ACSC rates. The literature suggests that the prevalence of international medical graduates is higher in poor, rural areas – and our findings suggest that these physicians are filling a need for primary care services there.

The last block of variables reflects market conditions. Medicare managed care penetration (MCPENE00) is associated with significantly but only slightly lower ACSC rates. The current commercial HMO penetration level (XHMO00) is negative but has no significant association, while current commercial PPO penetration (XPPO00) has a significant negative association – about 2 fewer admissions per standard deviation increase. Increase in commercial HMO penetration in the area (XHMODIF) has a significant negative association, while increase in commercial PPO penetration (XPPODIF) has a significantly positive association, about equal in magnitude (1.4 admissions per thousand, per standard deviation change). This may suggest collinearity, however, HMO and PPO penetration have not generally occurred in the same markets, and their simple correlation in 2000 was only 0.03. As a check for robustness, we dropped PPO variables to see whether the HMO variable coefficients were affected – they were not materially affected by these omissions, keeping their same sign and magnitude. However, when dropping both change variables (XPPODIF and XHMODIF) the beneficial effect of PPO relative to HMO penetration levels (XPPO00 relative to XHMO00) diminished. It is apparently important to distinguish the market entry effects with these change variables, because omitting this makes PPOs appear less effective than they actually are. The finding that PPO growth is positive to ACSC rates while HMO growth is negative suggests that PPOs may be entering markets where greater preventive care management is still needed, while HMOs are already established in markets with good managed care practices. There is a small but statistically significant negative association between market share of the top three group market commercial insurers (SHRLARGE3) and ACSC rates, about 1 fewer admission per standard deviation increase. Places with higher MediGap premiums have significantly higher ACSC rates – about 1.3 more admission per every $100 increase in premiums (MediGap enrollment rates in local areas are not available). Places with a greater prevalence of employer-sponsored coverage have significantly lower ACSC rates – a 6.8 percent higher prevalence is associated with about 1.3 fewer admission per thousand FFS beneficiaries in the area.

About 40 percent of the variation in ACSC rates among PCSAs is left unexplained by the model. This is likely due to the fact that some ACSC admissions are inevitable, even where there are high quality and timely primary health care services, either because health care resources are finite, and are therefore rationed, within as well as between all areas, or because patients are geographically mobile. Some patients with poorly managed ACSCs may move to other areas, taking their health conditions with them. Another consideration is that some preventive and primary health care required to prevent ACSC hospital admissions is provided by non-physicians such as: public health nurses, pharmacists, opticians, dentists, and health education professionals, among others. Because we did not have data to measure these additional providers, they are omitted from the analysis and as such, some of the remaining variation in ACSC admissions is left unexplained. There may also be unexplained cultural effects, such as living in cultural enclaves with unique health beliefs or behaviours. There may be effects from unmodeled physical barriers, such as living in swamps or bayous or in remote mountainous areas. Analysis of clustering in the large positive residuals from the model would suggest places where further investigation or targeted interventions might be appropriate.

## Discussion

This work presents an ecological model of spatial interaction among the elderly and their local environments. We use the model to explain the considerable variation in health care outcomes that exist among the elderly FFS Medicare population. The FFS population is the subgroup with the greatest latitude in choosing providers, and is composed of members across the income spectrum. This subgroup of the Medicare population also spans the urban-rural continuum and provides insights that cannot be gleaned from studying the Medicare managed care population, who are urban-based.

Our modeling includes demand, supply, and local environmental factors that serve to intervene along the pathways to health care utilization. Using a very local market definition based on Medicare patient flows to physicians, we sought to understand the relative importance of various factors that could impact preventive care utilization and result in unnecessary hospitalizations. We used admission rates for 11 ambulatory care sensitive conditions as the dependent variable, aggregated over three years within each of 6,455 primary care service areas. We employed good controls for person-specific demographics and disease severity, and were able to test several hypotheses regarding associations with other environmental variables.

Personal characteristics of the FFS beneficiaries explain almost half of the observed variation in ACSC rates, with factors such as dual eligibility status, octogenarian status, and disease risk explaining admissions of an order of magnitude comparable to a standard deviation in the ACSC rate distribution. About fifteen percent of the observed variation in ACSC rates can be explained by other factors included in the model.

Living in a community where the elderly were poor, or where they were rural, had no independent associations with ACSC admissions. However living in rural communities with greater proportions of poor elderly did have a significant positive association – ACSC admissions were about 3 higher with every standard deviation increase in the poor-rural interaction variable. And living in a sprawling place with longer average commute times also had a positive association with ACSC rates – about 3 higher per standard deviation increase in the proportion of workers who traveled more than 60 minutes daily to work. Thus living in rural poverty or in sprawling suburban places seem to have about equivalent impacts on access impediment for the elderly.

Managed care penetration appears to have a beneficial spillover effect – even though none of our sample population is in managed care *plans*, those who live in regions with higher managed care penetration exhibit lower admission rates for ACSCs. This suggests that there are beneficial spillovers from managed care presence onto medical practice styles in their markets, which is consistent with findings from an emerging literature. Because Medicare managed care plans have not penetrated rural areas, their beneficial spillovers are not available to the poor and rural elderly who seem to be the most vulnerable to shortages in preventive care services. The Medicare Modernization Act of 2003 includes legislation to stimulate entry of managed care into all regions of the U.S., so perhaps as time passes the poor-rural elderly disadvantage will diminish.

General physician availability does not seem to have a significant association with outcomes, while significant associations are found for other provider groups. In rural PCSAs with poor elderly populations and with proportionately more international medical graduates (IMGs) among the physician population, there were significantly fewer ACSC admissions. IMGs appear to provide needed services in these areas, reducing ACSC admissions by about 3 per thousand FFS beneficiaries (for a standard deviation increase in prevalence of IMGs). Non-physician clinicians also seem to provide needed primary care services, as the elderly who live in areas with more non-physician clinicians (relative to physicians) have significantly fewer ACSC admissions per thousand. A standard deviation increase in prevalence of non-physician clinicians is associated with about 2 fewer ACSC admissions.

## Conclusion

The relative importance of non-physician clinicians and international medical graduates in providing preventive care services to the elderly in those geographic places with greatest need can inform the ongoing debate regarding whether there is an impending shortage of physicians in the U.S. The current literature on physician supply is divided regarding whether there is either an existing or an upcoming shortage of physicians. Some argue that there is an upcoming shortage of physicians, based on current supply trajectories, demand and income growth estimates, and in consideration of the growing supply of non-physician clinicians [39-42]. Others maintain that there are persistent physician surpluses in many areas and that smoothing out physician settlement patterns may be all that is needed [24]. Our findings support the notion that non-physician clinicians and international medical professionals may be filling critical needs in geographic areas with the greatest shortage of physicians.

A recent study from the Community Tracking Survey found that, for the general population, residents in rural places seem to have comparable healthcare access to those residing in urban areas [28]. However, the findings in this paper suggest that what holds on average from survey data may not be true in all localities. Modeling the environmental conditions that intervene in health care utilization is important if we are to understand who uses healthcare and where. "Where" is important because interventions to improve average outcomes could be targeted to localities with the greatest observed need. Understanding what goes on at the micro level in places with the greatest need is an interesting and fruitful area for further medical geographic research.

## Methods

### Modeling spatial spillovers in preventive care utilization and outcomes

Social interaction has gained recognition as an important factor impacting health behaviors that put people at risk of adverse disease outcomes [43]. When people influence one another, neighborhoods will reflect similar underlying behaviors, and spatial clustering can occur in the behavioral risk factors and associated outcomes [36]. These sorts of social spillovers, also known as 'peer effects', are often difficult to capture with explanatory variables. However, omission of social spillovers that occur across geographic regions, i.e., neighborhood peer effects – can cause spatial correlation in other variables of interest, such as hospital utilization rates for ACSCs. If spatial autocorrelation is not accounted for properly in estimation, standard errors and/or coefficient estimates may be misleading [32].

There are, in fact, several difficult-to-measure factors that can impact patterns of utilization including efficacy in the healthcare system, such as physician practice styles or availability. These sorts of spillovers can be considered 'resource-based', resulting from investments in health infrastructure by one community that can have benefits for surrounding communities [44]. For example, luring another doctor to practice in a rural community may impact neighboring communities who are also in need of medical services. In this context, the impact of another doctor's availability on the community's ACSC rate depends on how stretched are her services to cover surrounding communities. The direct impact in the community of practice may be to reduce ACSCs, but there may also be an indirect effect in an adjacent community reducing ACSCs there. If PCSAs are perfectly defined, then these sorts of spillovers would not occur, as the boundaries would reflect self-contained physician markets.

Another factor that impacts spillovers is managed care's impact on medical care delivery in the regional market [45,46]. We include both Medicare managed care penetration and private sector managed care penetration in our model as factors reflecting the managed care climate. The only variables we cannot approximate and include as factors reflecting spillovers directly are actual health behaviors and/or the medical practice style prevalent in the local healthcare market. The geographic manifestation of these factors is expected to spill over from place to place.

We expect that specific practices and behaviors might spread to influence behaviors in nearby communities, so an empirical specification that captures these spillovers would be appropriate. To account for observed spillovers, we employ the spatial lag econometric model used by Anselin, Varga, and Acs [47] in their study of knowledge spillovers from universities to the private R&D sector. We are only aware of a few empirical studies that attempt to assess the extent of behavioral or practice style spillovers across health care markets, but a study by Lorant [48] found that failure to account for spatial interactions can lead to very misleading (overstated) estimates of the relationship between a factor (area socioeconomic status) and an outcome (mortality). In general, ignoring spatial spillovers can lead to biased and inefficient estimates of model parameters [32,33].

We also conduct an empirical test which confirms that the spatial dependence is more likely a spatial lag than a spatial error process. In a spatial error model, unobserved factors in neighboring areas are correlated leading to correlation in the error term across space. In a spatial lag model, observed outcomes are simultaneously determined with outcomes for neighboring areas, i.e., observations on the dependent variable are not independent due to behavioral spillovers. We conduct the specification test described in Anselin and Bera [49], p. 279, using Lagrange Multiplier test statistics on the OLS residuals, to determine whether the spatial dependence is more likely a spatial error or a spatial lag process. The test statistics are presented in Table 5, followed by an explanation regarding how we reached the conclusion that the lag process is more likely than the error process in these data.

**Table 5:** Diagnostics for Spatial Dependence: Lagrange Multiplier Tests for Error Versus LAG Dependence

**Symbol**	**Test**	**Degrees of Freedom**	**Test statistic value**	**p-value**
**RS_ρ_**	Lagrange Multiplier (lag)	1	1090.35	0.000
RS_ρ_*	Robust LM (lag)	1	173.32	0.000
**RS_λ_**	Lagrange Multiplier (error)	1	1002.00	0.000
RS_λ_*	Robust LM (error)	1	84.97	0.000

Methodology for proper diagnosis of error process in cross section: If neither **RS_ρ _**nor **RS_λ _**are significant, but robust tests (RS_ρ_* RS_λ_*) are, then ignore the robust tests. When **RS_ρ _**is more significant (lower p-value) than **RS_λ_**, and RS_ρ_* is significant while RS_λ_* is not, then lag autocorrelation is most likely the correct error structure. When **RS_λ _**is more significant (lower p-value) than **RS_ρ_**, and RS_λ_* is significant while RS_ρ_* is not, then error autocorrelation is most likely the correct error structure. We find that **RS_ρ _**is more significant then **RS_λ_**, as it has a larger test statistic value (and the same degrees of freedom). The same is also true of the robust tests, so we conclude that a lag process is more likely than an error process in these data.

In the context of our analysis, ACSC admission rates in one Primary Care Service Area (PCSA) are simultaneously determined with ACSC admission rates in adjacent PCSAs, through medical practice norms and health-seeking behaviors that impact the geographic manifestation of disease outcomes in the larger area (spanning 2 or more contiguous PCSAs). Observations on the dependent variable (ACSC admission rates by PCSA) are then not independent, as assumed under ordinary regression analysis.

**Figure 2:**
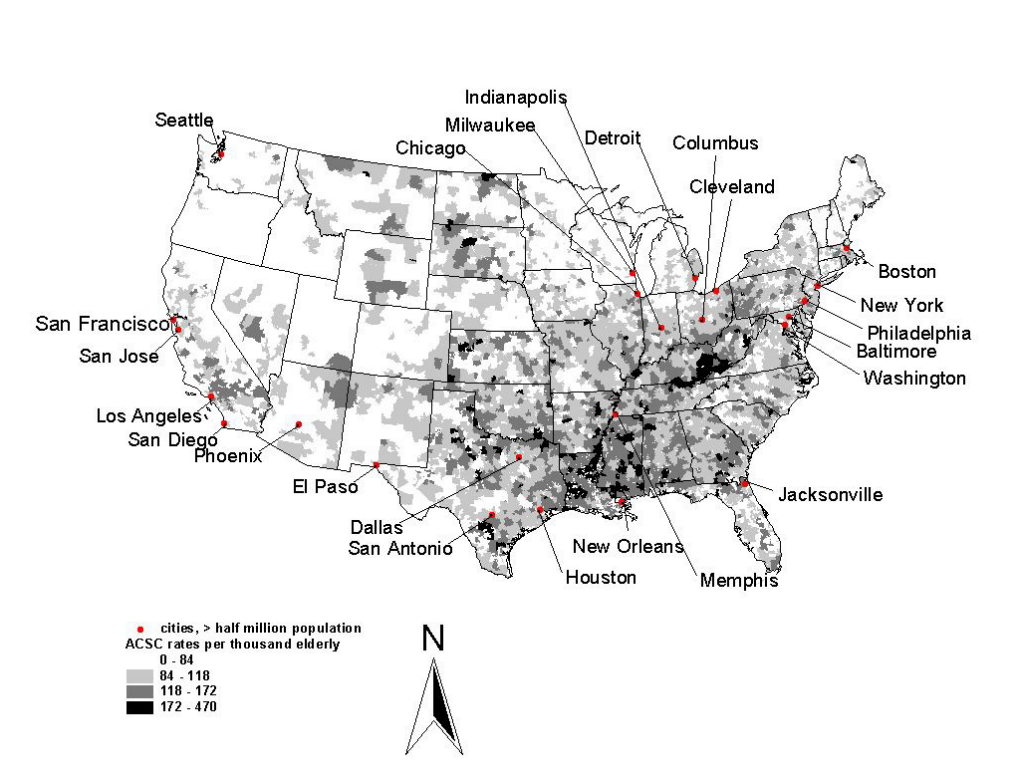
Spatial pattern of ACSC admission rates, 1998–2000, per thousand FFS beneficiaries in primary care service areas.

**Figure 3:**
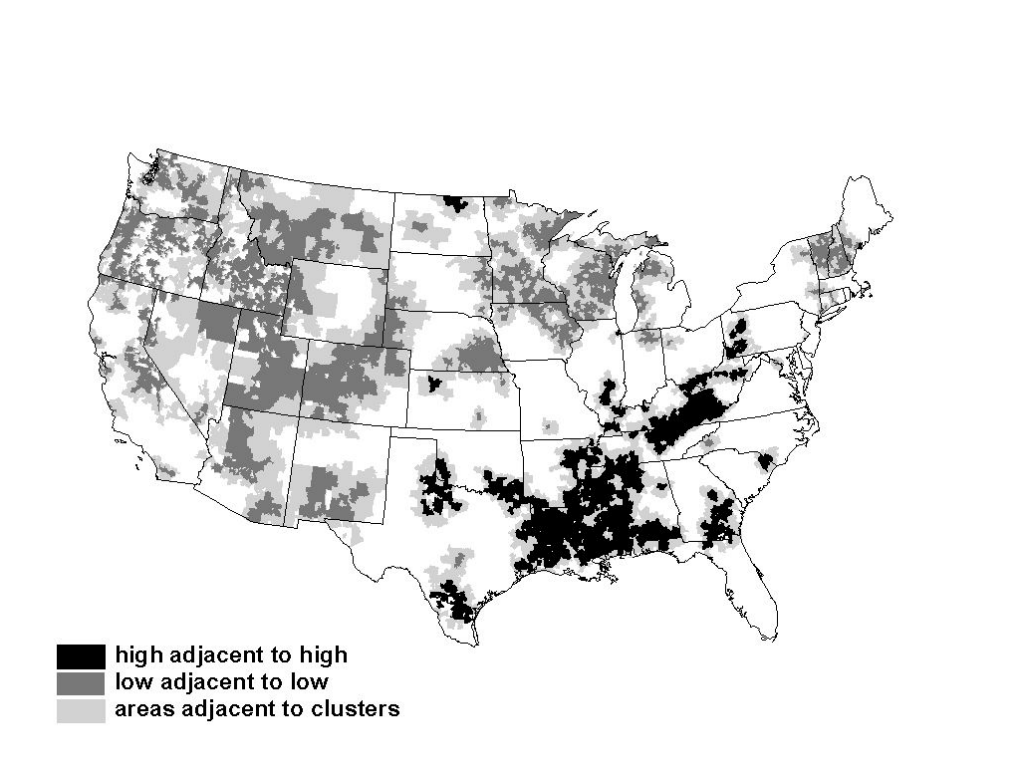
Spatial Clustering in ACSC Admission Rates, 1998–2000, Per Thousand FFS Beneficiaries in Primary Care Service Areas.

The raw ACSC rates are mapped in Figure 2. To test whether the rates are randomly distributed in space we employ a local Moran (LISA) test, with results presented in Figure 3. The hypothesis of spatial randomness is tested using a bootstrapping approach. The bootstrapping essentially compares the spatial autocorrelation in the variable of interest between a given PCSA and its contiguous PCSAs with that of the given PCSA and 999 spatially randomized sets of pseudo-neighbors. If the correlation among actual contiguous neighbors is far into the tail of the bootstrapped distribution derived from the 999 randomly chosen reference groups, then the degree of spatial autocorrelation is deemed significantly different than what could have occurred by chance [50]. The LISA test employed in Figure 3 uses a 1 percent significance level. We use a contiguity criterion which assumes all PCSAs with touching borders or corners are neighbors.

There are 6,475 PCSAs with elderly residents used in the analysis. PCSAs that are cluster centers (with all contiguous neighbors sufficiently similar to reject the hypothesis of spatial randomness) are depicted in the map, with positive clusters (high rates) shaded black and negative clusters (low rates) shaded dark grey. Areas contiguous to these cluster centers and contributing to the significant finding are shaded light grey. Figure 3 suggests that there is apparently considerable regionalization in the ACSC rates, so that adjacent PCSAs' rates are spatially correlated.

### Spatial regression

The descriptive analysis in Figure 3 is univariate. The aim of the spatial regression analysis is to combine multiple factors in a model which explains these spatial clusters. The form of the spatial lag model used in our regression accounts explicitly for the simultaneity in ACSC rates across neighboring locales. The model can be written:

ACSC = α + ρ*W_ACSC + β*X + ε     Equation 1

Where ACSC is the small-area rate of hospital admission for ambulatory care sensitive conditions per thousand FFS beneficiaries, defined for each of the 6,475 PCSAs used in the analysis. The parameter α is the constant, ρ is the spatial parameter reflecting the degree of spillovers among neighboring locales, W_ACSC is the average ACSC rate in neighboring locales, X is a vector of explanatory variables with parameter vector β, and ε is an identically and independently distributed normal error term. The model requires the specification of relevant neighbors for each PCSA, which we define as contiguous PCSAs (as in the LISA analysis of Figure 3). Ignoring the spatial variable and estimating the model using ordinary least squares may lead to an overstatement of the magnitude of the parameter vector β, to the extent that the spatial lag parameter ρ is statistically significant [33]. OLS coefficients would be inconsistent in the presence of an omitted spatial lag (such as the one specified in equation 1)[32]. The estimation of equation 1 requires either Maximum Likelihood or Instrumental Variables (IV) techniques because the spatial term W_ACSC is endogenous (simultaneously determined with ACSC on the LHS).

The Maximum Likelihood approach assumes that the ACSC model's errors are normally distributed, which is not likely to be true, because the ACSC rates distribution is quite skewed. The IV lag estimator is robust to skewness but requires large samples for statistical power. We provide in Table 4 results for models estimated by OLS, spatial lag with the Maximum Likelihood estimator, and spatial lag with an instrumental variables estimator, for comparative purposes. Before presenting the results we digress to discuss the data and methods, then what we expect to find based on some recent literature.

### Data and variable creation

We use the Primary Care Service Areas (PCSAs) provided by Health Resources and Services Administration as geographic health markets, built up from zip code tabulation areas (ZCTAs), reflecting flows of Medicare patients to primary care physicians. Our Medicare claims data has FFS beneficiaries resident in 6,475 of the 6,542 PCSAs. Twenty of these places are islands with no neighboring PCSAs, which we drop because observations with no neighbors are inappropriate for the spillovers model (Alaska and Hawaii were not included in the analysis). This leaves 6,455 contiguously arranged PCSA-level observations for the analysis. In the mainland U.S. (excluding Hawaii, Alaska, and the smaller islands) the mean land mass for a PCSA is about 485 square miles, with a mean population of about 44,751 persons. The PCSA areas are typically smaller than the 3,140 mainland U.S. counties, which have mean land mass of 1,142 square miles and mean population of 86,920. HRSA appended the PCSA files with over 900 additional variables aggregated to the PCSA level of geography. These variables include demographic and socio-economic data (U.S. Census Bureau), provider supply data (AMA/AOA), and Rural Health Clinics and Federally Qualified Health Centers (CMS, POS file). Tables 1 and 2 list the variables constructed for our analysis, and lists the original sources of the data for those variables.

In addition to the data provided by HRSA we acquired MEDPAR claims data aggregated by residential postal ZIP code for all Medicare beneficiaries age 65 and older, enrolled in Medicare FFS during 1998–2000 (about 74.9 million observations). There were 25.8 million claims for hospital admissions over this period, and about 7.2 million of these were admissions for ACSCs. Using these data we calculated the number of hospital admissions per PCSA, for any one of eleven ACSCs of particular interest for the elderly population [14]. We then aggregated the ACSC admissions over three years to construct market-level hospitalization rates (3-year ACSC rates defined for each PCSA market) to use as the outcome variable in our analysis.

Once the ZIP Code level data were aggregated and crosswalked with the ZCTA file, aggregation to the PCSA was straightforward. However, some of the intervening factors that we hypothesize will be important determinants of utilization describe Medicare Managed Care (MMC) penetration and entry and exit in MMC markets. Following the Balanced Budget Act of 1997, payments to Medicare managed care plans were reduced in many areas and many plans pulled out, which may have directly impacted beneficiaries in our sample if they were left stranded and returned to FFS Medicare. These MMC data are only available at the county-level because Medicare payments to MMC plans are county-specific. To use these county-level variables in our PCSA-level analysis, we considered how PCSAs are arranged relative to counties. Every county contributing to the PCSA's elderly population received a weight, and the MMC variables were created as a simple weighted average across all counties contributing to the PCSA.

## Competing interests

The author(s) declare that they have no competing interests.

## Authors' contributions

LM contributed to all aspects of the work: manuscript writing, data development, formulation of hypotheses, literature review, modeling, and estimation. ER contributed to data development, manuscript writing, modeling, and literature review. LA contributed to manuscript writing, modeling, and estimation. NG contributed to data development, manuscript writing, modeling, and estimation. JK contributed to data development, manuscript writing, modeling, and estimation.
